# Overexpression of *OLIG2* and *MYT1L* Transcription Factors Enhance the Differentiation Potential of Human Mesenchymal Stem Cells into Oligodendrocytes

**DOI:** 10.3390/cimb45050261

**Published:** 2023-05-07

**Authors:** Ifrah Fahim, Aisha Ishaque, Faiza Ramzan, Shamsul Azlin Bin Ahmad Shamsuddin, Anwar Ali, Asmat Salim, Irfan Khan

**Affiliations:** 1 Dr. Panjwani Center for Molecular Medicine and Drug Research, International Center for Chemical and Biological Sciences, University of Karachi, Karachi 75270, Pakistan; 2 Institute of Biological Sciences, Faculty of Sciences, Universiti Malaya, Kuala Lumpur 50603, Malaysia; 3 Department of Physiology, University of Karachi, Karachi 75270, Pakistan

**Keywords:** mesenchymal stem cells, fate specification, oligodendrocytes, differentiation, gene expression

## Abstract

Background: Demyelinating diseases represent a broad spectrum of disorders and are characterized by the loss of specialized glial cells (oligodendrocytes), which eventually leads to neuronal degeneration. Stem cell-based regenerative approaches provide therapeutic options to regenerate demyelination-induced neurodegeneration. Objectives: The current study aims to explore the role of oligodendrocyte-specific transcription factors (*OLIG2* and *MYT1L*) under suitable media composition to facilitate human umbilical-cord-derived mesenchymal stem cells (hUC-MSCs) differentiation toward oligodendrocyte for their potential use to treat demyelinating disorders. Methodology: hUC-MSCs were isolated, cultured, and characterized based on their morphological and phenotypic characteristics. hUC-MSCs were transfected with *OLIG2* and *MYT1L* transcription factors individually and in synergistic (*OLIG2* + *MYT1L*) groups using a lipofectamine-based transfection method and incubated under two different media compositions (normal and oligo induction media). Transfected hUC-MSCs were assessed for lineage specification and differentiation using qPCR. Differentiation was also analyzed via immunocytochemistry by determining the expression of oligodendrocyte-specific proteins. Results: All the transfected groups showed significant upregulation of *GFAP* and *OLIG2* with downregulation of *NES*, demonstrating the MSC commitment toward the glial lineage. Transfected groups also presented significant overexpression of oligodendrocyte-specific markers (*SOX10*, *NKX2.2*, *GALC*, *CNP*, *CSPG4*, *MBP*, and *PLP1*). Immunocytochemical analysis showed intense expression of OLIG2, MYT1L, and NG2 proteins in both normal and oligo induction media after 3 and 7 days. Conclusions: The study concludes that *OLIG2* and *MYT1L* have the potential to differentiate hUC-MSCs into oligodendrocyte-like cells, which is greatly facilitated by the oligo induction medium. The study may serve as a promising cell-based therapeutic strategy against demyelination-induced neuronal degeneration.

## 1. Introduction

Demyelinating diseases are associated with the gradual and progressive loss of myelin which ultimately results in the impairment of axonal conduction velocity and gives rise to various neurological complications [1]. Such disorders are further characterized by neuronal degeneration [2]. The most representative primary demyelinating disease includes multiple sclerosis (MS). It is a chronic autoimmune disorder of the CNS that is characterized by the formation of demyelinating plaques within the white matter. It is strongly associated with the initiation of inflammatory cascades, and the ultimate damage to the neuronal axons. Worldwide prevalence of MS is expected to be 2.8 million by the year 2020, affecting 1 out of every 2786 individuals and diagnosed at an average age of 32 years [3,4].

Although continuous advances have been made over time for pharmacological therapies, these approaches provide transient treatment and only offer symptom management [5]. The majority of medications focus on one of the two strategies, i.e., minimizing disease progression or treating a specific symptom. However, these medications are frequently associated with undesirable side effects with a limited therapeutic window because of the protective role of the blood–brain barrier [6]. Immunomodulatory therapies are most successful in the early disease phase but are not effective for relapsing remitting MS [7]. Consequently, researchers have been working to develop more effective treatments by introducing gene-therapy approaches by following three steps: (i) prevention of specific symptoms, (ii) reversing disease progression, and (iii) healing CNS damage through facilitating remyelination and axonal repair [6]. Recently, clinically relevant biomarkers for MS, such as miRNAs, have emerged and can be utilized to evaluate the efficacy of ongoing treatment, detect pathophysiological processes, and develop personalized treatment plans [8].

One of the emerging and effective approaches for the treatment of various degenerative anomalies is regenerative medicine. Cell-based therapy is an important aspect of regenerative medicine and is the most applicable treatment modality [2,3,9,10]. Stem cell-based treatment is a promising therapeutic strategy that effectively facilitates tissue repair and performs cellular replacement of the damaged area. Mesenchymal stem cells (MSCs) are adult, multipotent stem cells that have been reported to treat various degenerative pathologies, including neurodegenerative and demyelinating disorders [10,11,12,13,14]. A study previously conducted by our group has shown the differentiation potential of MSCs toward neuronal lineage [15]. MSCs have also been reported to induce oligodendrocyte differentiation under the influence of various factors. A study conducted by Oppliger et al. reported that Wharton’s jelly-derived MSCs secrete proangiogenic and neuroprotective factors, which regulate oligodendrocyte differentiation [14]. MSCs have also been found to be effective in reducing the myelin sheath damage caused by spinal cord injury when co-transplanted with neural stem cells [16]. Zhang et al. demonstrated that hBM-MSCs treatment in experimental autoimmune encephalomyelitis (EAE) mice greatly reduced the area of demyelination and facilitated the increase in BDNF+ cells [17]. Phases 1 and 2 of the clinical trials are also being conducted using MSCs to analyze their therapeutic effect in MS patients. A study submitted to clinicaltrials.gov indicated the safety and feasibility of MSCs injection (both intrathecally and intravenously) in patients with MS [18]. Genetic manipulation of MSCs is a potential strategy for enhancing their therapeutic potential and induction of fate specification by the overexpression of certain transcription factors or genes [19,20,21]. *OLIG2* belongs to the basic helix-loop-helix transcription factor family and is a key regulatory gene for oligodendrocyte lineage specification and regulates crucial phases of early oligodendrocyte development. It has also been documented as an upstream *SOX10* regulator, which also plays a key role to regulate oligodendrocyte development [20,22]. *MYT1L* belongs to the family of myelin transcription factors and is involved in the proliferation and differentiation of oligodendrocyte precursor cells, which essentially performs myelination and remyelination of the central nervous system. It has also been reported to play an important role in neurogenesis and neural differentiation. *MYT1L* significantly improves myelination [19], suggesting a possible therapeutic target for myelin repair.

Keeping in consideration the potential role of *OLIG2* and *MYT1L* in oligodendrocyte differentiation and myelination, respectively, this study was designed to induce hUC-MSC differentiation toward oligodendrocyte by their genetic modification, both in normal and oligo induction media. The study may provide a promising cell-based treatment option for demyelination-induced neurodegenerative diseases.

## 2. Materials and Methods

### 2.1. Ethical Consent and Umbilical Cord Sample Collection

Human umbilical cord samples (*n* = 10) were collected in a sterile glass bottle containing PBS and 0.5% EDTA (Sigma-Aldrich Chemie, GmbH, Taufkirchen, Germany) from Zainab Panjwani Memorial Hospital, following the cesarean section delivery of healthy donors. Informed consent was taken from the donors’ parents. The study protocol regarding the human participants was approved by the independent ethical committee (reference no. IEC-009-UCB-2015) of the Dr. Panjwani Center for Molecular Medicine and Drug Research, International Center for Chemical and Biological Sciences, University of Karachi.

### 2.2. Isolation and Propagation of hUC-MSCs

The cord tissue was washed thoroughly with PBS to remove blood clots and was cut into small pieces of about 1–3 mm^2^. The cord explants were transferred into T-75 tissue culture flasks (Cat. No. 708003, Nest, Wuxi, China) containing Dulbecco’s modified Eagle’s medium (DMEM) (Cat. No. 11965-092, Gibco, Paisley, UK) supplemented with 10% fetal bovine serum (FBS) (Cat. No. 10500-64, Gibco, Paisley, UK), 1 mM sodium pyruvate (Cat. No. 11360-070, Gibco, UK), 1 mM L-glutamine (Cat. No. 25030681, Gibco, Paisley, UK), and 100 U antibiotic (penicillin–streptomycin) (Cat. No. 15070-063, Gibco, Paisley, UK). The flasks were incubated at 37 °C in a 5% humidified CO_2_ incubator (ESCO, Singapore). The non-adherent hematopoietic cells were removed from the flasks by replacing the media with fresh DMEM after every 3 days. The cells migrated out from the explants, adhered to the flask surface, and started to proliferate. At this stage, MSCs were termed as passage 0 (P0) cells. When the cells reached 80–90% confluence, they were subcultured to subsequent passages using 0.25% trypsin–EDTA solution (Cat. No. 25200-056, Gibco, Paisley, UK). Passages 2–4 hUC-MSCs were used for all the experimental work.

### 2.3. Characterization of hUC-MSCs

hUC-MSCs were characterized based on their morphological features, surface-specific markers by immunocytochemistry and flow cytometry, and by assessing their multilineage differentiation potential as described in our previous studies [23,24].

#### 2.3.1. Morphological Features of hUC-MSCs

To assess morphological features, hUC-MSCs were maintained for 3–5 subsequent passages and routinely examined under phase contrast microscope. Images were captured from each passage using a charge-coupled device (CCD) camera (Nikon Eclipse Ts2, Tokyo, Japan).

#### 2.3.2. Immunocytochemistry

Passage 2 hUC-MSCs were seeded on coverslips placed in a 24-well plate (CLS3526, Corning, Corning, NY, USA) at a density of about 3000–5000 cells/well. Complete DMEM was added in each well and the plate was incubated at 37 °C in a humidified CO_2_ incubator. The next day, the media were removed and cells were washed twice with PBS. Cells were fixed with 4% paraformaldehyde (PFA) (Cat. No. 30525894 Carl Roth, Karlsruhe, Germany) and permeabilized with 0.1% Triton-X 100 (Cat. No. 9036195, Sigma-Aldrich, Taufkirchen, Germany) for 10 min each at room temperature. Non-specific binding sites were blocked using 2% blocking solution (2% BSA with 0.1% Tween 20 in PBS). Primary antibodies against MSC positive markers (CD29, CD105, Vimentin, CD117, Lin28, and Stro1) and negative markers (CD45 and HLA-DR) at a dilution of 1:200 were added in the designated wells and incubated overnight at 4 °C. The next day, antibody solutions were removed and cells were washed gently five times with PBS. Alexa fluor-546 conjugated secondary antibody at a dilution of 1:200 and Alexa fluor-488 phalloidin was added in each well and incubated for 1 h at 37 °C. Nuclei were stained with DAPI (0.5 µg/mL) for 15 min at room temperature. Cells were washed five times with PBS and coverslips were mounted on glass slides with Fluoromount aqueous mounting medium (F4680, Sigma, St. Louis, MI, USA). Images were captured under a fluorescence microscope (NiE, Nikon, Tokyo, Japan). Antibody details are mentioned in Table 1.

#### 2.3.3. Immunophenotyping

hUC-MSCs were cultured in T-75 flask and trypsinized when 80–90% confluence was attained. Pellet was washed with PBS and incubated with blocking solution (1% BSA, 1 mM EDTA, and 0.1% sodium azide) for 30 min. The suspension was centrifuged at 400 g for 5 min and primary antibodies for MSC-specific markers (anti CD90, CD105, CD44, and CD73) were added for 2 h at 37 °C. The cell suspension was centrifuged at 400 g for 5 min, washed 3 times with ice-cold FACS solution, and incubated with Alexa flour 546 conjugated secondary antibody for an hour at 37 °C. Finally, the suspension was centrifuged again and washed three times. The pellet was resuspended in ice-cold FACS solution and analyzed by flow cytometer (BD FACS Calibur, Becton Dickinson, Franklin Lakes, NJ, USA).

#### 2.3.4. Trilineage Differentiation

To assess trilineage differentiation potential, hUC-MSCs were seeded in a 6-well plate and nourished with DMEM. Once the cells reached 70–80% confluence, DMEM was replaced with lineage-specific induction media, i.e., osteogenic (0.1 μM dexamethasone (Cat. No. D4902, Sigma-Aldrich, Taufkirchen, Germany), 10 μM β-glycerophosphate (Sigma-Aldrich; Cat. No. G6376), and 50 μM L-ascorbate 2-phosphate (Cat. No. A8960, Sigma-Aldrich, Taufkirchen, Germany), adipogenic (1 μM dexamethasone, 10 μM insulin (Cat. No. 11070738, Sigma-Aldrich, Taufkirchen, Germany), 200 μM indomethacin (Cat. No. 190217, MP Biomedical, Burlingame, CA, USA), chondrogenic (100 nM dexamethasone, 20 ng TGFβ1 (Cat. No. RPA124Hu01, Claud Clone, Wuhan, China), 10 ng insulin, and 100 μM ascorbic acid (Sigma-Aldrich; Cat. No. 50817). The induction media were changed after every 3 days. After 21 days of incubation, cells were washed with PBS and fixed with 4% PFA. MSC differentiation was analyzed by staining the cells with Alizarin red (Cat. No. 155984, MP Biomedical, Burlingame, CA, USA), Oil red O (Cat. No. 130223, Sigma-Aldrich, Taufkirchen, Germany) and Alcian blue (Cat. No. 74240, Carl Roth, Karlsruhe, Germany) stains for the presence of calcium deposits, oil droplets, and proteoglycans, respectively. Stained cells were observed under bright-field microscope (Eclipse TE 2000-S, Nikon, Tokyo, Japan).

### 2.4. Isolation of Plasmid Vectors

*E. coli* stab cultures of *OLIG2* and *MYT1L* plasmid constructs were obtained from Addgene (www.addgene.org, accessed on 13 March 2023); *OLIG2* plasmid ID # 32933 and *Myt1L* plasmid ID # 32926). *E.coli* were grown on Luria broth and plasmid DNA was isolated by using GeneJET™ Plasmid Maxiprep Kit (Thermo Scientific, Waltham, MA, USA) according to the instructions provided by the manufacturer. Isolated plasmids were quantified via spectrophotometric analysis and electrophoresed on 1% agarose gel to evaluate their purity.

### 2.5. Experimental Groups

The study comprised four experimental groups, i.e., control (non-transfected hUC-MSCs), *OLIG2*-transfected, *MYT1L*-transfected, and synergistic (*OLIG2* + *MYT1L*)-transfected groups. All the groups were incubated under two different media compositions, i.e., normal DMEM and oligo induction medium (5% FBS supplemented OPTI-MEM, 0.1 mM β-mercaptoethanol, and B-27 supplement-2x). The experimental details have been mentioned in Table 2.

### 2.6. Transfection of hUC-MSCs

Lipofectamine-based transfection technique was used to transfect hUC-MSCs with the desired transcription factors (*OLIG2* and *MYT1L*). Briefly, Lipofectamine^®^ 3000 transfection reagent (Cat. No. L3000-008, Thermo Fisher Scientific, Waltham, MA, USA) and master mix of each plasmid DNA were diluted individually in OPTI-MEM. Diluted plasmids were mixed with lipofectamine (1:1 ratio) and incubated for 15 min at room temperature. This DNA/lipid mixture was added dropwise in 80–90% confluent T-75 flasks individually and in combination. The control group was not transfected with any of the transcription factors. Each transfected group was incubated under standard cell culture conditions for 3 and 7 days in two different media compositions, as mentioned in Table 2.

### 2.7. Morphological Examination of Transfected hUC-MSCs

Transfected hUC-MSCs were incubated in normal and oligo induction media for 3 and 7 days and monitored regularly for the induction of differentiation by observing their morphological features under phase contrast microscope.

### 2.8. Gene Expression Analysis

RNA was extracted from control and transfected groups using Trizol reagent (15596026, Invitrogen) reagent and quantified via spectrophotometer. RNA yield equivalent to 1 µg was reverse-transcribed into cDNA using RevertAid First Strand cDNA Synthesis Kit (K1622, Thermo Scientific, USA) according to the manufacturer’s instructions. Real-time PCR was performed to analyze the expression of oligodendrocyte-specific genes in the transfected groups. Each sample was run in triplicates and GAPDH was used as a standard internal control. Primer sequences specific to each gene have been provided in Table 3.

### 2.9. Protein Expression Analysis of Transfected MSCs

Normal and transfected MSCs incubated in both normal and oligo induction media were analyzed for the expression of oligodendrocyte-specific proteins (OLIG2, MYT1L, NG2, and MBP) by immunocytochemistry as mentioned above. Images were captured under fluorescent microscope (Nikon, Tokyo, Japan). The fluorescent intensity of each group was quantified via ImageJ software.

### 2.10. Statistical Analysis

Statistical analysis was performed using IBM SPSS Statistics software (version 21; SPSS Inc, Armonk, NY, USA) to establish statistical significance at the accepted level; *p* ≤ 0.05 (*p* < 0.05 = *, *p* < 0.01 = **, and *p* < 0.001 = ***). Each experiment was run in triplicates and acquired data were analyzed through an independent sample *t*-test. The results have been presented as mean ± SEM.

## 3. Results

### 3.1. Umbilical Cord Processing; Isolation, Propagation, and Morphological Features of hUC-MSCs

**Figure 1:**
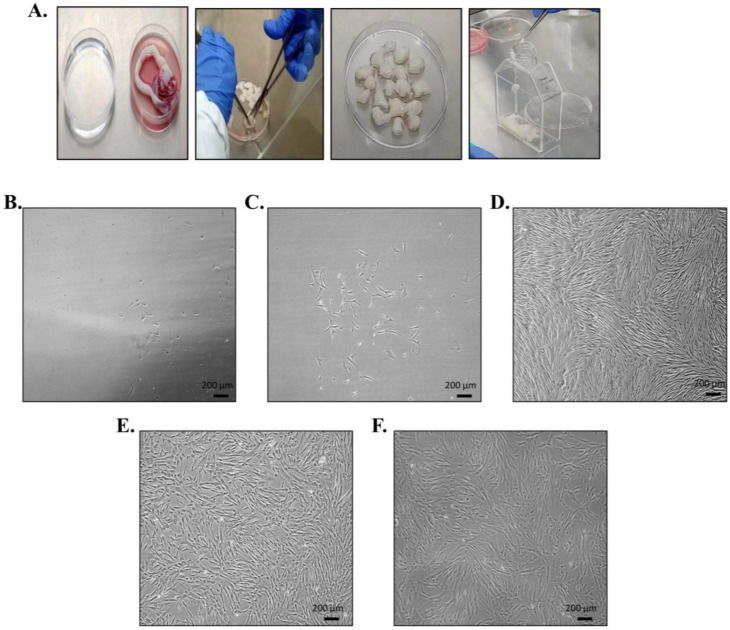
Umbilical cord processing; hUC-MSC isolation and propagation: hUC-MSCs were isolated from human umbilical cord. (**A**) Cord tissue was washed thoroughly, minced into small pieces (explant) and transferred to T-75 tissue culture flasks for incubation. (**B**) Cord tissue shows cell outgrowth after about 2 weeks of explant culture and (**C**) proliferating colonies of hUC-MSCs. (**D**) P0 confluent hUC-MSCs presenting fibroblast like morphology, which were subcultured to (**E**,**F**) passage 1 and passage 2, respectively.

Umbilical cord tissue was processed under the sterile environment of a biosafety cabinet *(*Figure 1A). After about 2 weeks of explant culture, MSCs migrated out of the tissues, adhered to the flask surface, and started to form colonies. The cells proliferated further and adopted fibroblast like morphology, which is the typical characteristic of MSCs. At this stage, hUC-MSC culture was termed P0. On reaching 80–90% confluence, they were subcultured to subsequent passages (P1 and P2) for experimental work (Figure 1B–F).

### 3.2. Characterization of hUC-MSCs

**Figure 2:**
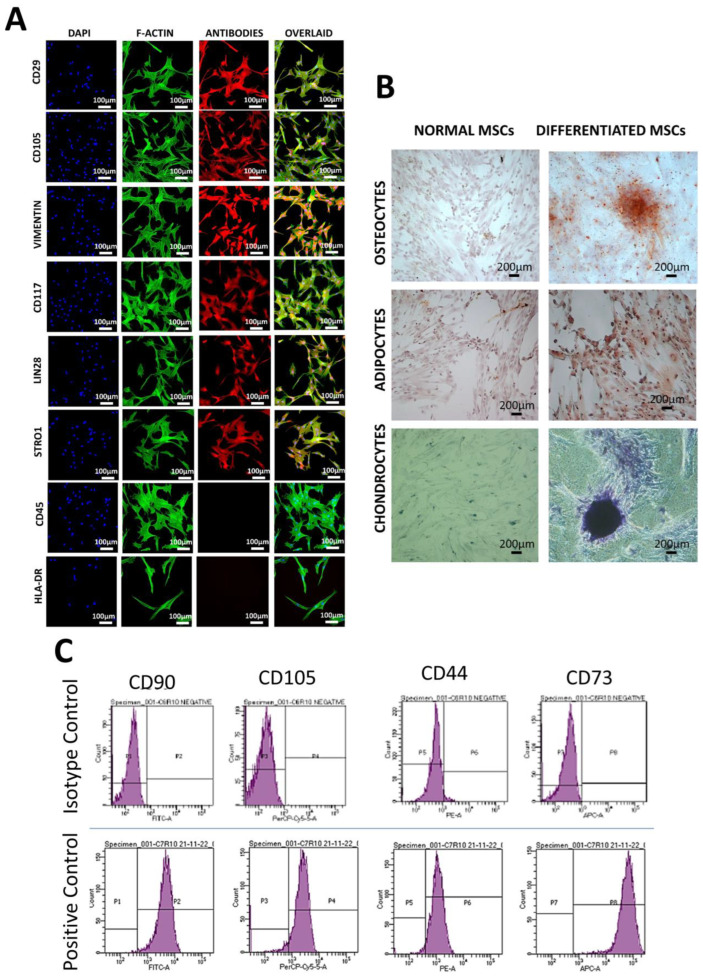
Characterization of mesenchymal stem cells: (**A**) hUC-MSCs showing positive expression of surface-specific markers, i.e., CD29, CD105, Vimentin, CD117, Lin28, and Stro1 and negative expression of hematopoietic markers, i.e., CD45 and HLA-DR. (**B**) Bright field images showing hUC-MSC differentiation toward osteogenic, adipogenic, and chondrogenic lineages indicated by the presence of mineral deposits, oil droplets, and proteoglycan content, stained with Alizarin red, Oil red O, and Alcian blue stain, respectively. (**C**) Flow cytometry analysis presenting the positive expression of MSC-specific markers, i.e., CD44, CD73, CD90, and CD105.

hUC-MSCs characterization was performed via immunocytochemistry, flow cytometry, and trilineage differentiation potential. Immunocytochemical analysis indicated the positive expression of MSC-specific markers (CD29, CD105, Vimentin, CD117, Lin28, and Stro1) and negative expression of hematopoietic markers (CD45 and HLA-DR) as shown in Figure 2A. hUC-MSCs also showed prominent features of trilineage differentiation, i.e., mineral deposits in case of osteogenic differentiation, oil droplet formation, and proteoglycan content in case of adipogenic and chondrogenic differentiation after staining with Alizarin red, Oil red O and Alcian blue stains, respectively (Figure 2B). Flow cytometry also revealed the positive expression of MSC surface antigens (CD90, CD105, CD44, and CD73) as shown in Figure 2C.

### 3.3. Transfection, Differentiation, and Morphological Assessment of hUC-MSCs

hUC-MSCs were transfected with *OLIG2* and *MYT1L* transcription factors individually and in synergistic (*OLIG2* + *MYT1L*) groups. Transfected cells started to show slight morphological features of oligodendrocyte-like cells after 3 days of incubation in the oligo induction medium. On the seventh day of incubation, hUC-MSCs showed pronounced differentiation toward oligodendrocyte-like cells, as indicated by their morphological characteristics. Differentiation was more evident in transfected hUC-MSCs incubated in the oligo induction medium compared to normal medium.

### 3.4. Gene Expression Analysis of Transfected hUC-MSCs Incubated in Normal Medium

**Figure 3:**
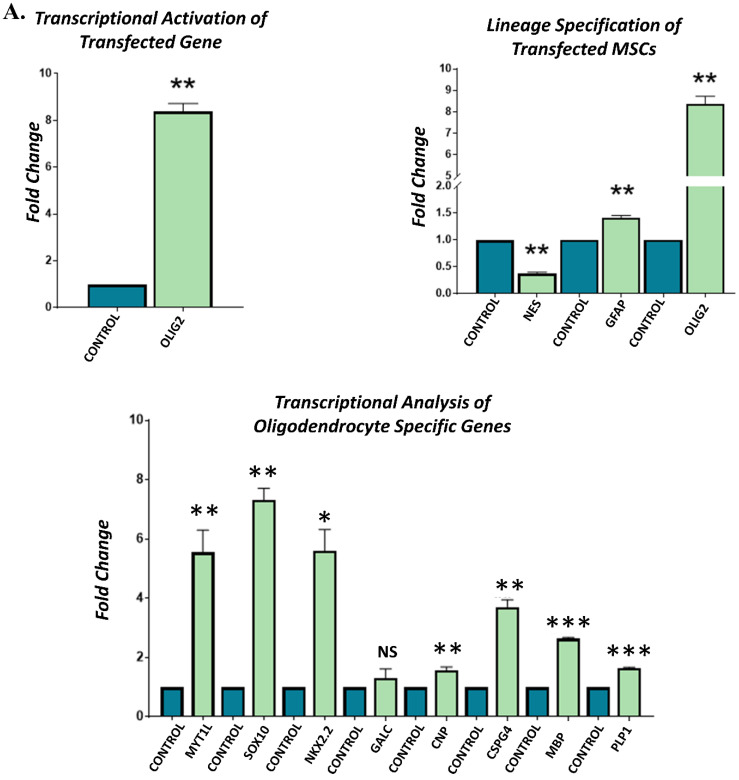

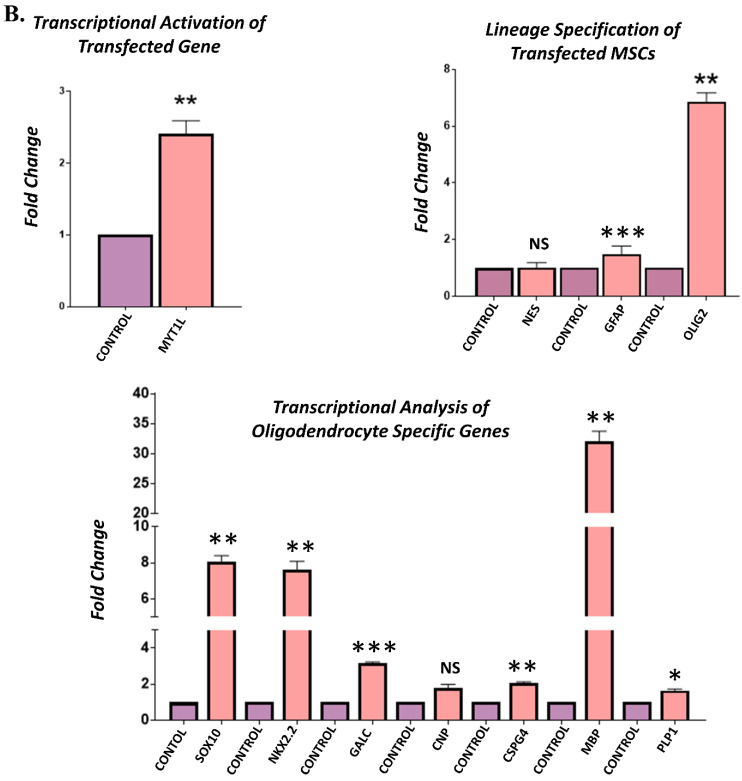

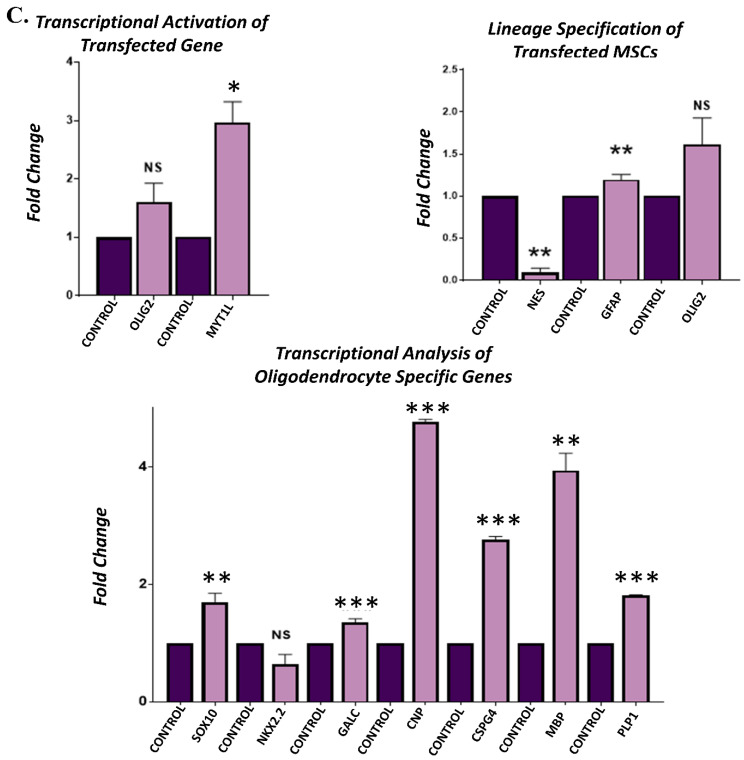
Gene expression analysis of transfected hUC-MSCs incubated in normal medium: qPCR analysis indicating the transcriptional activation of *OLIG2* and *MYT1L* genes in (**A**) *OLIG2*, (**B**) *MYT1L*, and (**C**) synergistic (*OLIG2 + MYT1L*) transfected groups. Lineage specification analysis presented higher expression levels of *GFAP* and *OLIG2* in comparison to *NES*, demonstrating the glial fate specification. Transcriptional analysis of oligodendrocyte-specific genes, i.e., *SOX10*, *NKX2.2*, *GALC*, *CNP*, *CSPG4*, *MBP*, and *PLP1*, presenting their significant overexpression compared to the non-transfected control group after 3 days of incubation in normal medium. Each experiment was run in triplicates and acquired data were analyzed through an independent sample *t*-test at statistical significance level; *p* ≤ 0.05 (*p* < 0.05 = *, *p* < 0.01 = **, and *p* < 0.001 = ***). NS = not significant.

Gene expression analysis of the *OLIG2*, *MYT1L*, and their synergistic *(OLIG2 + MYT1L)* transfected groups showed significant transcriptional activation of *OLIG2* and *MYT1L* genes in their respective groups compared to non-transfected control after 3 days of incubation in the normal medium. Lineage specification analysis indicated significant downregulation of *NES* and upregulation of *GFAP* and *OLIG2* genes, showing the commitment of transfected hUC-MSCs toward glial lineage. Transcriptional analysis of oligodendrocyte-specific genes revealed the substantial overexpression of *SOX10*, *NKX2.2*, *GALC*, *CNP*, *CSPG4*, *MBP*, and *PLP1*, indicating the differentiation and fate specification of hUC-MSCs toward oligodendrocyte-like cells. However, *NKX2.2* was found to be downregulated in the synergistic group (Figure 3).

### 3.5. Gene Expression Analysis of Transfected hUC-MSCs Incubated in Oligo Induction Medium

**Figure 4:**
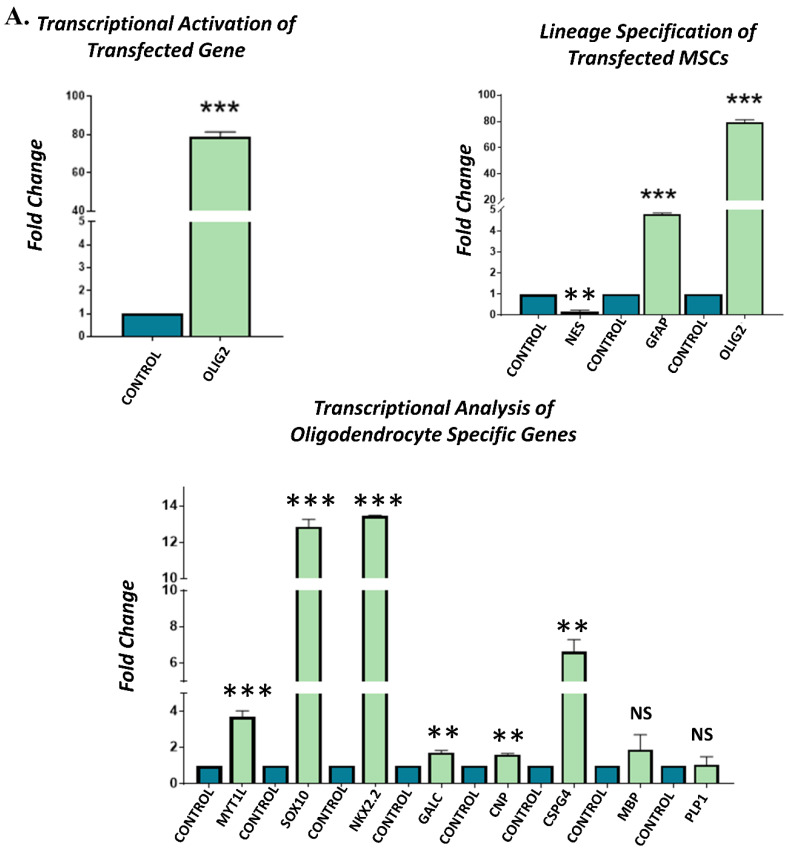

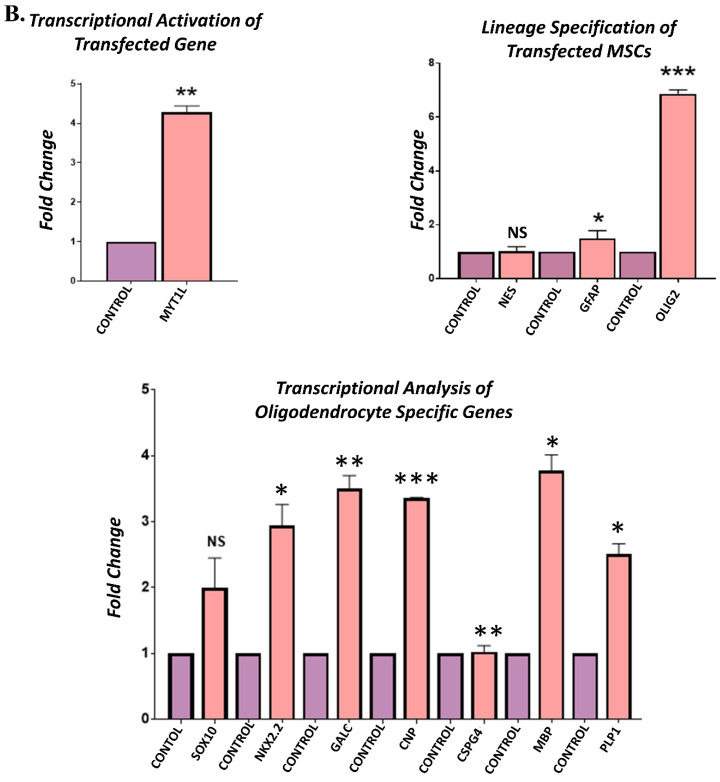

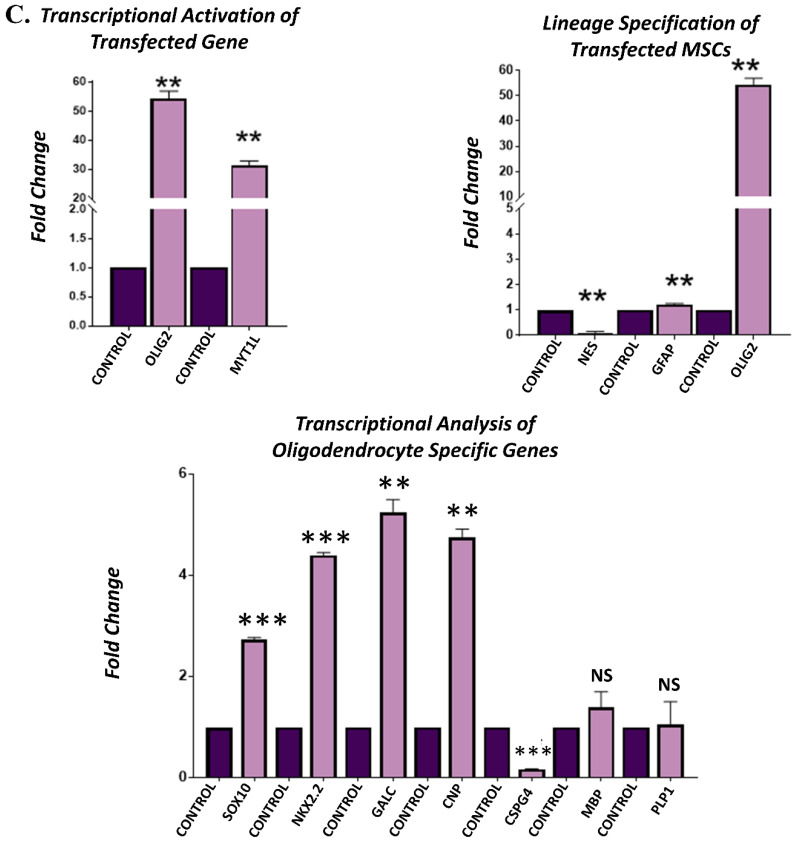
Gene expression analysis of transfected hUC-MSCs incubated in oligo induction medium: qPCR analysis showing the transcriptional activation of *OLIG2* and *MYT1L* genes in (**A**) *OLIG2*, (**B**) *MYT1L*, and (**C**) synergistic (*OLIG2 + MYT1L*) transfected groups. Lineage specification analysis indicated higher expression level of *GFAP* and *OLIG2* compared to *NES*, demonstrating the glial fate specification. Transcriptional analysis of oligodendrocyte-specific genes, i.e., *SOX10*, *NKX2.2*, *GALC*, *CNP*, *CSPG4*, *MBP*, and *PLP1*, presenting their significant overexpression compared to the control group after 3 days of incubation in oligo induction medium. Each experiment was run in triplicates and acquired data were analyzed through an independent sample *t*-test at statistical significance level; *p* ≤ 0.05 (*p* < 0.05 = *, *p* < 0.01 = **, and *p* < 0.001 = ***). NS = not significant.

Gene expression analysis of the *OLIG2*, *MYT1L*, and their synergistic *(OLIG2 + MYT1L*) transfected groups showed significant transcriptional activation of *OLIG2* and *MYT1L* genes in their respective groups compared to non-transfected control, after 3 days of incubation in the oligo induction medium. Lineage specification analysis of all the transfected groups indicated significant downregulation of *NES*; however, *GFAP* and *OLIG2* genes were significantly overexpressed, showing the commitment of transfected hUC-MSCs toward glial lineage. Transcriptional analysis of oligodendrocyte-specific genes revealed the upregulation of *SOX10*, *NKX2.2*, *GALC*, *CNP*, *CSPG4*, *MBP*, and *PLP1*, demonstrating the fate specification and differentiation of hUC-MSCs toward oligodendrocyte-like cells. However, *CSPG4* exhibited reduced expression in the synergistic *(OLIG2 + MYT1L*) group as compared to the control group (Figure 4).

### 3.6. Protein Expression Analysis of Transfected hUC-MSCs in Normal and Oligo Induction Media after 3 Days of Incubation

**Figure 5:**
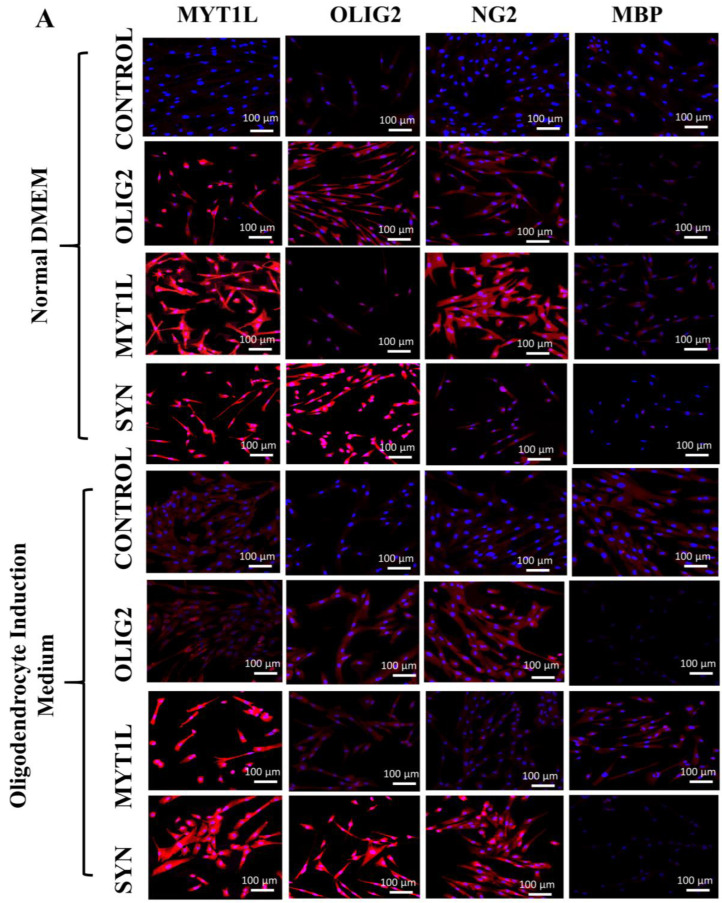

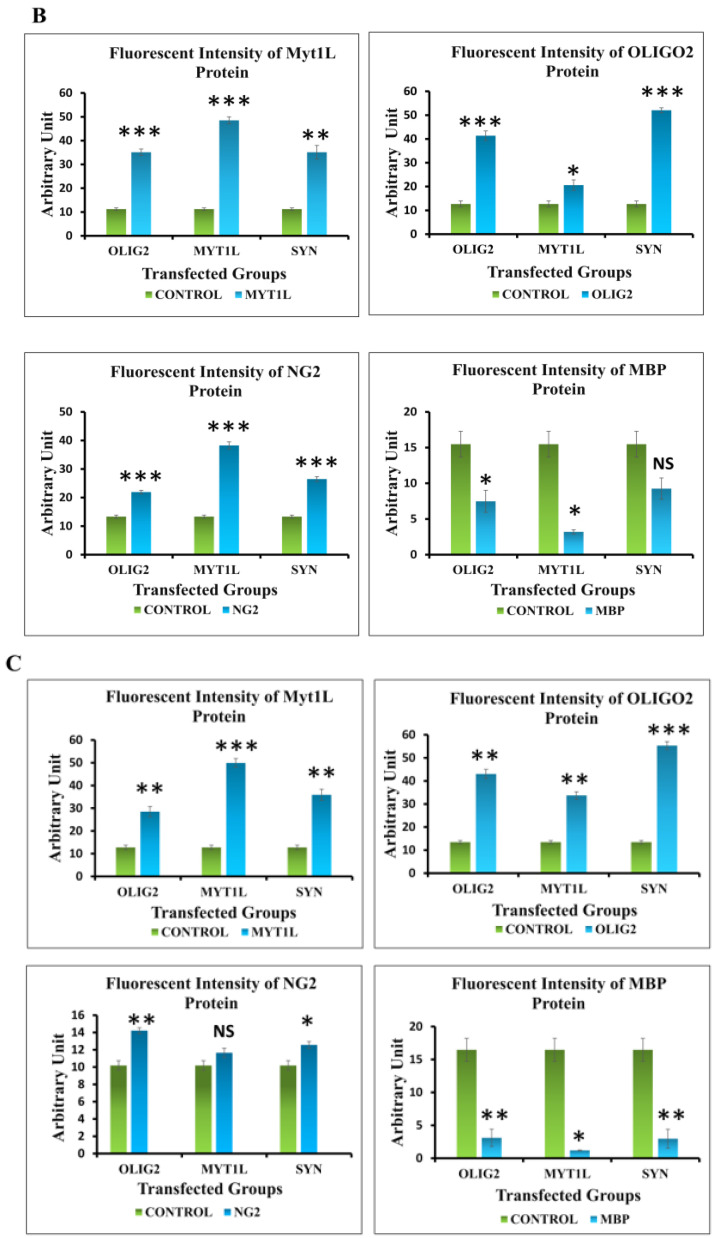
Protein expression/immunocytochemical analysis of transfected hUC-MSCs in normal and oligo induction media after 3 days: (**A**) Immunocytochemical analysis of *OLIG2*, *MYT1L*, and synergistic (*OLIG2* + *MYT1L*) transfected groups showing intense expression of MYT1L, OLIG2, and NG2, and a reduced expression of MBP proteins, incubated in both normal and oligo induction media. (**B**,**C**) Fluorescent intensities showing intense expression of *MYT1L*, *OLIG2*, and NG2 proteins, whereas MBP was found to exhibit reduced intensity in all the transfected groups compared to the non-transfected control group after 3 days of incubation in both normal and oligo induction media. The fluorescent values (*n* = 30) were analyzed through an independent sample *t*-test at statistical significance level; *p* ≤ 0.05 (*p* < 0.05 = *, *p* < 0.01 = **, and *p* < 0.001 = ***). NS = not significant.

Immunocytochemical analysis indicated the positive expression of MYT1L, OLIG2, and NG2 and negative expression of MBP proteins in *OLIG2*, *MYT1L*, and their synergistic (*OLIG2 + MYT1L*) transfected groups after 3 days of incubation in both normal and oligo induction media as shown in Figure 5A. Fluorescent signal exhibited by MYT1L, OLIG2, and NG2 proteins also showed a significant increase in intensities in all the transfected groups incubated in the normal medium after 3 days. However, MBP showed reduced intensity compared to the control group (Figure 5B). *OLIG2*, *MYT1L*, and their synergistic (*OLIG2* + *MYT1L*) transfected groups also showed intense fluorescent signal of MYT1L, OLIG2, and NG2 and reduced signals for MBP proteins after 3 days of incubation in the oligo induction medium compared to control group (Figure 5C).

### 3.7. Protein Expression Analysis of Transfected hUC-MSCs in Normal and Oligo Induction Media after 7 Days of Incubation

**Figure 6:**
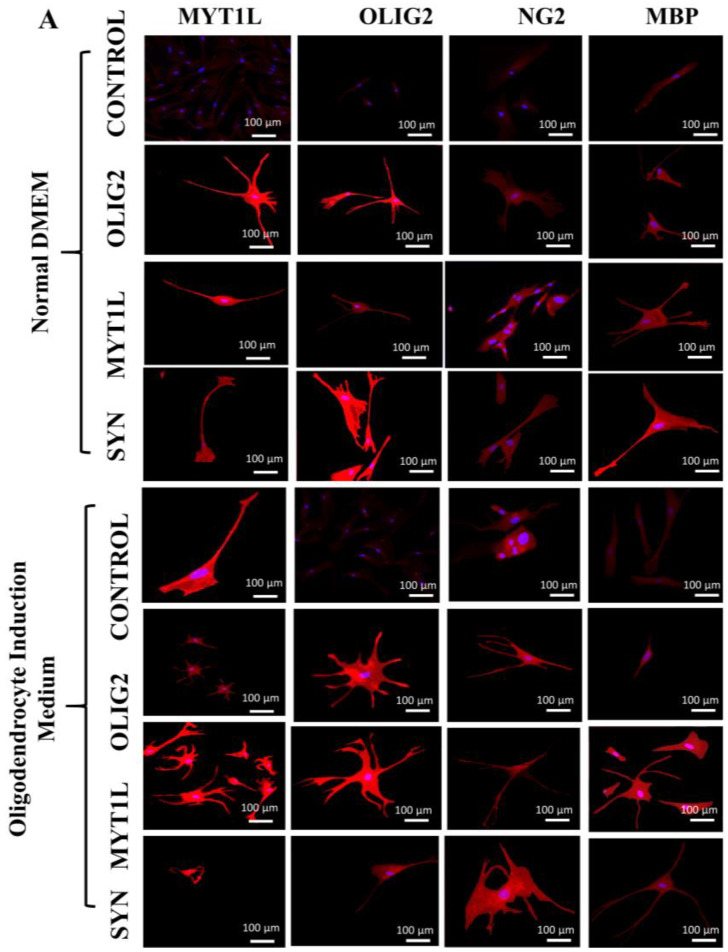

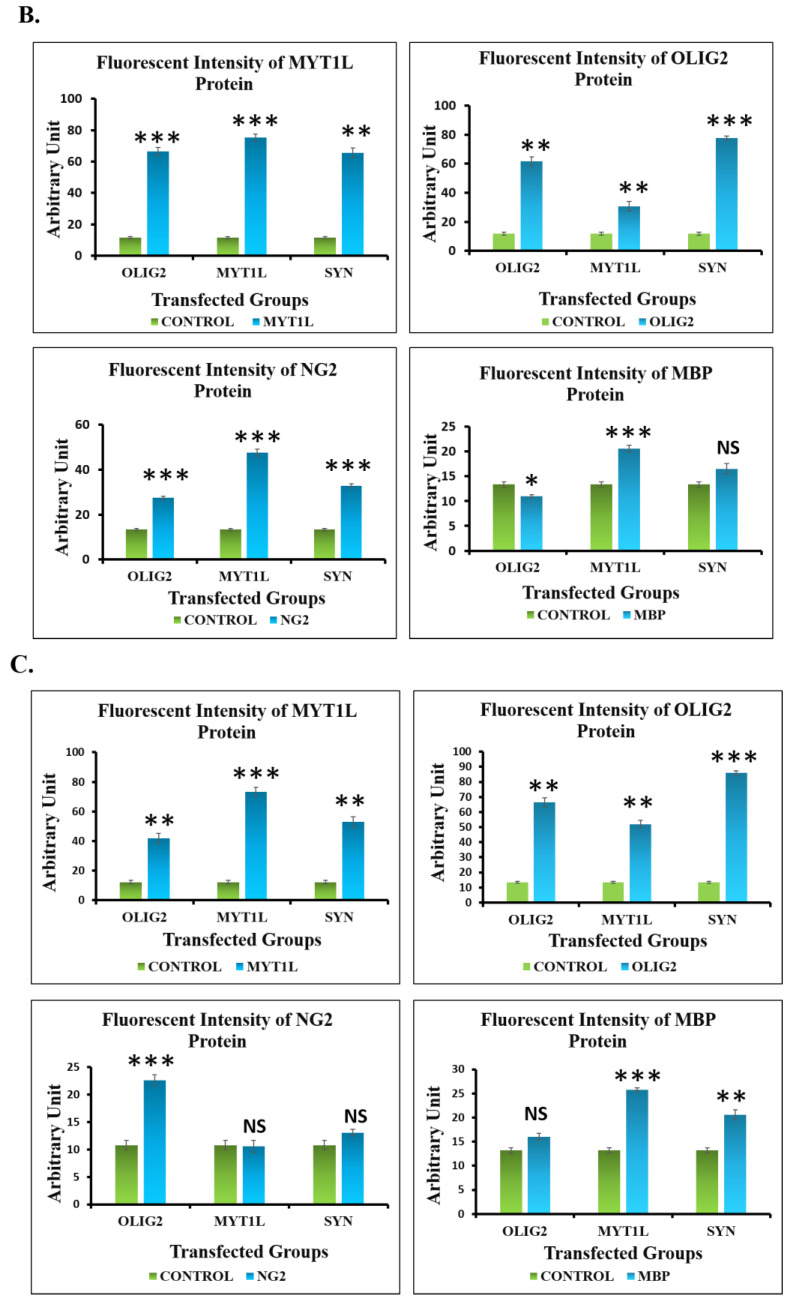
Protein expression/immunocytochemical analysis of transfected hUC-MSCs in normal and oligo induction media after 7 days: (**A**) Immunocytochemical analysis of *OLIG2*, *MYT1L*, and synergistic (*OLIG2* + *MYT1L*) transfected groups, indicating positive expression of MYT1L, OLIG2, NG2, and MBP proteins. (**B**,**C**) Fluorescent intensities showing intense expression of oligodendrocyte-specific proteins, i.e., MYT1L, OLIG2, NG2, and MBP in all the transfected groups as compared to the non-transfected control group after 7 days of incubation in both normal and oligo induction media. However, the OLIG2-transfected group exhibited reduced fluorescence signals of MBP in the normal medium. The fluorescent values (*n* = 30) were analyzed through an independent sample *t*-test at statistical significance level; *p* ≤ 0.05 (*p* < 0.05 = *, *p* < 0.01 = **, and *p* < 0.001 = ***). NS = not significant.

Immunocytochemical analysis indicated the positive expression of oligodendrocyte-specific proteins, i.e., MYT1L, OLIG2, NG2, and MBP by differentiated hUC-MSCs in all the transfected, i.e., *OLIG2*, *MYT1L*, and synergistic (*OLIG2* + *MYT1L*) groups after 7 days of incubation in normal and oligo induction media, as shown in Figure 6A. Fluorescent signals of MYT1L, OLIG2, NG2, and MBP proteins also showed a significant increase in intensities in all the transfected groups in the normal medium after 7 days of incubation, except the *OLIG2*-transfected group, which showed a reduced MBP intensity (Figure 6B). *OLIG2*, *MYT1L*, and their synergistic (*OLIG2* + *MYT1L*) transfected groups showed an intense expression of MYT1L, OLIG2, NG2, and MBP proteins after 7 days of incubation compared to the control group in the oligo induction medium (Figure 6C).

## 4. Discussion

The current study demonstrates the effective role of transcriptional regulators *(OLIG2* and *MYT1L)* as a remarkable tool for stimulating myelin repair in lethal demyelinating disorders using cell-based therapeutic approach. Such disorders are often characterized by the loss of oligodendrocytes (OLs). These cells play a fundamental role to provide metabolic support to axons and are responsible for the appropriate conduction of nerve impulse; therefore, an injury or damage to OLs result in neurodegeneration [1]. OLs and their progenitor cells (OPC) are the potential promising targets for cell-based regenerative applications due to their less diversified functional features and greater region/subtype specificity compared to neurons [3,25].

Presently, there is no effective treatment available for MS. Current pharmacological therapies only offer symptom management and slow down the disease progression by modulating the immune response or inflammatory cascades [26].

Therefore, there is a need to find treatment strategies that can facilitate endogenous myelination, thus favoring neuroprotection. Recent studies have reported the effective role of cell-based therapies for neurological and demyelinating disorders. In this context, mesenchymal stem cells are the attractive candidate due to their multilineage differentiation toward cells of all lineages, i.e., ectoderm, endoderm, and mesoderm. MSCs have also been reported to exhibit remarkable regeneration potential, self-renewal capabilities, and immunomodulatory features [2,12]. MSCs are also effective to treat neurological disorders and exhibit neuroprotective features which make them an attractive candidate for cell-based therapies [20,26].

A study conducted on BM-MSCs-derived oligodendrocyte precursor cells (OPCs) was found to boost remyelination and reduce demyelination after their differentiation into mature oligodendrocytes in animal models [27]. hUC-MSCs have also been shown to improve behavioral functions and reduce the histopathological deficits in EAE mice over a long term (i.e., 50 days) by inhibiting perivascular immune cell infiltrations and reducing demyelination and axonal injury in the spinal cord [28]. A recently conducted study on Wharton’s jelly-derived MSCs has shown their enhanced remyelination potential and increased oligodendrocyte count in a cuprizone-induced MS model [29].

In the present study, hUC-MSCs were isolated from the human umbilical cord and characterized based on their native features, i.e., presence of surface-specific markers and trilineage differentiation potential. Our preliminary study was categorized into two main parts to assess the effect of two different media compositions, i.e., normal and oligo induction media (5% FBS-supplemented OPTI-MEM, 0.1 mM β- mercaptoethanol, and B-27 supplement-2x) on MSC fate specification and differentiation via their genetic modification by inserting oligodendrocyte-specific transcription factors, i.e., *OLIG2* and *MYT1L*. To achieve this ultimate objective, *OLIG2* and *MYT1L* plasmids were used to transfect hUC-MSCs individually and in synergistic *(OLIG2 + MYT1L*) groups, through a non-viral gene transfer method, using Lipofectamine^®^ 3000 transfection reagent. Non-viral gene expression of the transcription factors has been reported to serve as a powerful transfection strategy that facilitates the stable delivery of transcription factors and allows their transcriptional activation [18].

Transfected cells were incubated in normal and oligo induction media and observed for differentiation by examining their morphological features. We observed the induction of differentiation on day 3 with no apparent change in morphology; however, hUC-MSCs after 7 days of incubation in the oligo induction medium indicated prominent cell differentiation with characteristic morphological features of oligodendrocyte-like cells. The differentiation potential of MSCs toward oligodendrocytes has also been reported previously by other studies [22].

Gene expression profile of transfected hUC-MSCs showed transcriptional activation of *OLIG2* and *MYT1L* genes in their respective transfected groups, indicating the induction of differentiation. To assess the fate specification of transfected hUC-MSCs, lineage-specific markers were analyzed, which revealed the downregulation of neuronal stem cell marker (*NES)* and upregulation of glial lineage markers, i.e., *GFAP* and *OLIG2.* Higher expression of *OLIG2* gene compared to *GFAP* demonstrates the fate specification of MSCs toward oligodendrocytes.

MSC differentiation was further assessed by analyzing the expression of oligodendrocyte-specific markers (*SOX10*, *NKX2.2*, *GALC*, *CNP*, *CSPG4*, *MBP*, and *PLP*1). Expression of these genes was found to be significantly upregulated in all the transfected groups and it was more pronounced in the oligo induction medium in comparison to the normal medium, demonstrating the differentiation and fate specification of MSCs toward oligodendrocytes. Expression of these OL markers demonstrates the differentiated state of transfected cells. Overexpression of *SOX10* and *OLIG2* has also been reported previously to induce oligodendrocyte differentiation, thereby acting as the master regulatory genes. *OLIG2* and *NKX2.2* have also been shown to support the development of OPC lineage, and *Myt1L* expression by mature oligodendrocytes is associated with myelination and remyelination [30,31].

We also determined the protein expression by immunocytochemistry. The differentiated hUC-MSCs significantly expressed oligodendrocyte-specific proteins, i.e., OLIG2, MYT1L, and NG2 after 3 and 7 days of incubation in normal and oligo induction media, as indicated by their fluorescent intensities. Fluorescent signals were found to be significantly higher in the oligo induction medium as compared to the normal medium. Taken together these findings, our study demonstrates the fundamental role of *OLIG2* and *MYT1L* transcription factors in hUC-MSCs differentiation toward oligodendrocyte-like cells, which were greatly facilitated by the oligo induction medium, emphasizing the significance of transcriptional regulators as a remarkable tool for stimulating myelin repair in lethal demyelinating disorders. Further studies are required to explore the mechanism behind oligodendrocyte differentiation and to analyze their myelination potential in animal models of demyelinating disorder. The findings of the study may serve as a promising cell-based therapeutic modality in treating demyelinating and neurodegenerative ailments.

## 5. Conclusions

The overall findings of our study conclude that *OLIG2* and *MYT1L* play a crucial role to induce hUC-MSC differentiation toward oligodendrocyte-like cells in both normal and oligo induction media at gene and protein levels. However, hUC-MSC differentiation and fate specification were greatly facilitated by the oligo induction medium. The study emphasizes the role of the transcription regulator as a remarkable tool for stimulating myelin repair in lethal demyelinating disorders. These findings may help to develop cell-based therapeutic strategies for demyelinating diseases and their use in future clinical studies.

## Figures and Tables

**Table 1:** List of antibodies used for MSC characterization and oligodendrocyte differentiation.

S. No.	Primary Antibody	Function/ Binding to	Working Dilution	Catalog Number	Manufacturer
	MSC Characterization Markers				
1.	CD29	Membrane glycoprotein	1:100	MAB-1981	Chemicon International, Katy, TX, USA
2.	CD105	Endoglin	1:100	560839	BD Pharmingen, San Diego, CA, USA
3.	Vimentin	Epithelial–mesenchymal transition	1:100	V6389	Sigma-Aldrich, Inc., St. Louis, MI, USA
4.	C-Kit (CD117)	Stem cell factor receptor	1:100	32–9000	Zymed Laboratories, Inc., South San Francisco, CA, USA
5.	Lin28	Cell surface *MSC marker*	1:100	PA1-096	Molecular Probes, Invitrogen, Eugene, OR, USA
6.	Stro-1	Mesenchymal precursor cell marker	1:100	14-6688-82	Molecular Probes, Invitrogen, Eugene, OR, USA
7.	HLA-DR	MHC class II immunogenic marker	1:100	14-9956-82	Molecular Probes, Invitrogen, Eugene, OR, USA
8.	CD45	Lymphocyte antigen	1:100	CBL415	BD Pharmingen, Diego, CA, USA
	Oligodendrocyte-specific Markers				
9.	OLIG2	Oligodendrocyte lineage-specific marker	1:100	PA5-85734	Molecular Probes, Invitrogen, Eugene, OR, USA
10.	Myt1L	Myelin transcription factor 1-like	1:50	PA5-34468	Molecular Probes, Invitrogen, Eugene, OR, USA
11.	NG2	Neural/Glial antigen 2	1:100	PA5-100235	Molecular Probes, Invitrogen, Eugene, OR, USA
12.	MBP	Myelin basic protein	1:100	MA1-24990	Molecular Probes, Invitrogen, Eugene, OR, USA
	Secondary Antibodies				
13.	Goat Anti-rabbit	Alexa Fluor 546	1:200	A-11010	Molecular Probes, Invitrogen, Eugene, OR, USA
14.	Anti-Rat IgG Isotype	Alexa Fluor 488	1:200	012-090-003	Jackson Immuno Research, Inc., West Grove, PA, USA

**Table 2:** Experimental setup details of the hUC-MSC genetic modification.

Experimental Groups	Transcription Factor(s) Inserted
Normal Medium	
Control	--
*OLIG2*-transfected	*OLIG2*
*MYT1L*-transfected	*MYT1L*
Synergistic	*OLIG2* + *MYT1L*
Oligo Induction Medium	
Control	--
*OLIG2*-transfected	*OLIG2*
*MYT1L*-transfected	*MYT1L*
Synergistic	*OLIG2* + *MYT1L*

**Table 3:** Primer sequences of the targeted genes used for qPCR analysis.

	Genes	Primer Sequences (5′-3′)	Annealing Temperature
Lineage-specific Genes	*NES* (F) *NES* (R)	TTCCAGACTCCACTCCCCTG CTCAGTCCCCAGGTCCTCAA	55 °C
	*GFAP* (F) *GFAP* (R)	ATGCTGGCTTCAAGGAGACC GGTGGCTTCATCTGCTTCCT	55 °C
	*OLIG2* (F) *OLIG2* (R)	TCAAGTCATCCTCGTCCAGC TCACCAGTCGCTTCATCTCC	55 °C
Oligodendrocyte-specific Gene	*MYT1L* (F) *MYT1L* (R)	GACTGCGGAACAGGATTTGG CGACCAGGGTTTGAAGATGC	55 °C
	*NKX2.2* (F) *NKX2.2* (R)	TTCCTCGCCACCAGCC TTCGGCCACAGAGCCC	55 °C
	*SOX10* (F) *SOX10* (R)	ACGTCAAGCGGCCCAT TCCCACCTTGCTCGGC	55 °C
	*GALC* (F) *GALC* (R)	GAATTTTCCAAAGAATGGCTGGG CAGTGATGATCAAGTTACTGCCA	55 °C
	*CNP* (F) *CNP* (R)	CCTTCAAGAAGGAGCTGCGA AGCTTGTCCACATCACTCGG	55 °C
	*CSPG4* (F) *CSPG4* (R)	GGATGCCACCCTACAAGTGA TTTTGCGCCTCTAGTGGGAT	55 °C
	*PLP1* (F) *PLP1* (R)	ATTCTTTGGAGCGGGTGTGT GAAGGTGAGCAGGGAAACCA	55 °C
	*MBP* (F) *MBP* (R)	GCGGCCCCTGTCTCC GCGGCTCCCTGGGTC	55 °C

## Data Availability

No additional data to provide.
